# High levels of lipoprotein(a) are associated with a lower prevalence of diabetes with advancing age: Results of a cross-sectional epidemiological survey in Gran Canaria, Spain

**DOI:** 10.1186/1475-2840-11-81

**Published:** 2012-07-02

**Authors:** Mauro Boronat, Pedro Saavedra, Nuria Pérez-Martín, María J López-Madrazo, Carlos Rodríguez-Pérez, Francisco J Nóvoa

**Affiliations:** 1 Section of Endocrinology and Nutrition, Hospital Universitario Insular, Avda. Marítima del Sur, s/n.,, 35016, Las Palmas de Gran Canaria, Spain; 2 Department of Medical and Surgical Sciences, University of Las Palmas de Gran Canaria, Las Palmas de Gran Canaria, Spain; 3 Department of Mathematics, University of Las Palmas de Gran Canaria, Las Palmas de Gran Canaria, Spain

**Keywords:** Lipoprotein(a), Diabetes, Insulin resistance, HOMA-IR, Age

## Abstract

**Background:**

Recent data suggest that concentrations of lipoprotein(a) [Lp(a)] may be inversely associated with the risk of diabetes. This study analyzed the relationships between Lp(a) and both diabetes and insulin resistance in an adult cohort from the island of Gran Canaria, Spain.

**Methods:**

Lp(a), homeostasis model assessment for insulin resistance (HOMA-IR) and conventional risk factors for diabetes were assessed in a sample of 1,030 adult individuals participating in a cross-sectional population-based epidemiological survey in the city of Telde. Diabetes was defined according to the WHO 1999 criteria, or as a previous diagnosis of diabetes. To identify patients at risk for diabetes, an Lp(a) cutoff level of 46 mg/dl was selected previously using classification and regression tree analysis. A multivariate logistic regression model with L_2_-regularization was used to assess the independent effect of Lp(a) on diabetes and its interactions with variables traditionally linked to the disease. Additionally, to investigate the effect of Lp(a) on insulin resistance, a parametric model was developed to describe the relationship between age and HOMA-IR values in subjects with levels of Lp(a) ≤46 or >46 mg/dl.

**Results:**

Along with variables known to be associated with diabetes, including age, mean blood pressure, serum triglycerides, and an interaction term between age and low HDL cholesterol, the logistic model identified a significant inverse association for diabetes and the interaction term between age and Lp(a) levels >46 mg/dl. According to the proposed parametric model, HOMA-IR was significantly lower in subjects of all ages who had Lp(a) levels >46 mg/dl.

**Conclusions:**

These results suggest that the age-related increase in the probability of having diabetes is significantly lower in subjects with Lp(a) levels >46 mg/dl. This could be explained in part by a lower insulin resistance in this subset of the population.

## Background

Lipoprotein(a) [Lp(a)] is a plasma lipoprotein consisting of a LDL-like particle with a molecule of apolipoprotein B100 covalently linked to a very large additional glycoprotein known as apolipoprotein(a). Elevated Lp(a) levels constitute an independent risk factor for cardiovascular disease in the general population [1].

Lp(a) concentrations show considerable variation in healthy individuals, and are controlled mainly by genetics [2,3]. Nearly 90% of Lp(a) variation depends on biosynthesis of the distinctive apolipoprotein(a) protein, which is encoded by the LPA locus [2]. Many classic and novel risk factors have been investigated to determine whether they explain the higher risk of cardiovascular disease among people with diabetes [4-6]. In particular, several studies have examined the possibility that type 2 diabetes could influence Lp(a) concentrations. The results of several small case–control studies have been controversial. Subjects with diabetes have been found to have higher [7,8], similar [9] or lower levels of Lp(a) [10] than controls without diabetes. Using a different approach to analyze the interaction between Lp(a) and diabetes, a more recent prospective study [11] of healthy US women aged 45 years or older (Women’s Health Study [WHS]) revealed an inverse association between Lp(a) and the risk of incident type 2 diabetes. The authors replicated their findings in a Danish population-based cohort (Copenhagen City Heart Study [CCHS]) with prevalent diabetes. These findings suggest that Lp(a) has opposite effects on the risks of cardiovascular disease and diabetes, increasing the former and decreasing the latter.

The aim of the present study was to examine the relationships between Lp(a) concentrations and both diabetes and insulin resistance in a cohort of adult subjects from the general population of the Canary Islands, Spain.

## Research design and methods

### Subjects and measurements

The study population was composed of 1,030 adult subjects (≥30 years-old) who were participating in a cross-sectional population-based study in Telde, a city located on the island of Gran Canaria, Canary Islands, Spain. The design of this survey was described previously [12]. Participants were selected randomly from the local census. All subjects provided written informed consent for participation in the study, which was approved by the ethics committee of the Hospital Universitario Insular of Las Palmas.

Subjects filled out a survey questionnaire, underwent physical examination, gave fasting blood samples, and, except for those with known diabetes, underwent a 75-g standardized oral glucose tolerance test (OGTT). Diabetes was defined as a previous diagnosis of diabetes with ongoing treatment with oral agents and/or insulin or was defined using the 1999 WHO diagnostic criteria (fasting plasma glucose ≥7.0 mmol/l and/or 2-h plasma glucose ≥11.1 mmol/l) [13]. A total of 128 subjects were classified as having type 2 diabetes, while 902 did not have diabetes. The homeostasis model assessment for insulin resistance (HOMA-IR) was calculated according to the formula HOMA-IR = fasting insulin (mU/l) × fasting plasma glucose (mmol/l)/22.5 [14]. Plasma insulin was measured by a chemiluminescent assay (Elecsys 2010; Roche Diagnostics, Basel, Switzerland) and Lp(a) was measured by nephelometry, using a Dimension RxL autoanalyzer (Dade-Behring, Liederbach, Germany).

### Statistical analysis

A classification and regression tree (CART) analysis selected an Lp(a) cutoff level of 46 mg/dl for the identification of patients at risk for diabetes. CART is a nonparametric validated statistical procedure in which classification and regression trees are constructed to predict an outcome variable from among a large number of variables and their interactions. Explanatory variables can be categorical and/or numeric [15]. To analyze the eventual effect of Lp(a) on diabetes, a multivariate logistic analysis was carried out. Variables entered into the model were Lp(a) (with categories >46 and ≤46 mg/dl), age, sex, presence of abdominal obesity (defined as a waist circumference ≥102 cm for men and ≥88 cm for women) [16], blood pressure (entered as mean arterial pressure [MAP]), low levels of HDL cholesterol (HDL cholesterol <40 mg/dl for men and <50 mg/dl for women) [16], serum triglycerides (logarithmic scale), LDL cholesterol, and self-reported use of statins. Levels of triglycerides were logarithmically transformed to reduce skewness. All variables in the groups with or without diabetes were checked for outliers. In order to explore all possible interactions, we used a variant of logistic regression with L_2_-regularization to fit models with interactions [17]. A forward selection procedure of variables and their interactions was performed based on the Akaike’s information criterion. The model was fitted using the iteratively reweighted ridge regressions algorithm. Odd-ratios of interest were computed from the obtained model along with estimated 95% confidence intervals.

To further explore glucose homeostasis based on levels of Lp(a), the relationship between age and HOMA-IR values (in log scale) was modeled as follows:

(1) $$\text{E }[\text{log}(\text{HOMA}-\text{IR})|\text{Lp}\left(\text{a}\right)]=\theta +\beta \times \text{ age }+\gamma \times \text{Lp}{\left(\text{a}\right)}_{46}\times {\text{ age}}^{2}\text{.}$$

In this parametric equation, Lp(a)_46_ has a value of 1 if Lp(a) is greater than 46 mg/dl and a value of 0 if Lp(a) is equal to or less than 46 mg/dl. All statistical analyses were performed using the R-package stepPlr.

## Results

Table 1 shows the demographic characteristics of the survey population. The median level of Lp(a) was 14.0 (interquartile range: 5.1–29.8) mg/dl in subjects with diabetes and 13.8 (6.0–33.0) mg/dl in subjects without diabetes (*p* = 0.35). There was no significant difference in the percentage of individuals with Lp(a) >46 mg/dl between the groups with or without diabetes (9.4% vs. 14.3%; *p* = 0.13). A significantly higher percentage of subjects with diabetes reported taking statins compared to subjects without diabetes (29.7% vs. 14.8%; *p* < 0.001).

**Table 1:** Characteristics of the population according to the presence or absence of diabetes

	**Total (N = 1030)**	**Diabetes (N = 128)**	**No diabetes (N = 902)**	***p*-value**
Age (years)	48.0 ± 11.9	58.9 ± 10.4	46.5 ± 11.3	<0.001
Male / Female (%)	43.5 / 56.5	57.8 / 42.2	41.5 / 58.5	<0.001
BMI (kg/m^2^)	28.2 ± 5.0	31.0 ± 5.4	27.8 ± 4.8	<0.001
Waist (cm)	97 ± 14	107 ± 12	95 ± 13	<0.001
Abdominal obesity (%)	57.4	83.6	53.7	<0.001
Systolic blood pressure (mmHg)	119 ± 16	134 ± 16	117 ± 15	<0.001
Diastolic blood pressure (mmHg)	73 ± 10	79 ± 10	72 ± 10	<0.001
Smoking (%)	24.2	11.7	25.9	<0.001
Current use of statins (%)	14.8	29.7	12.6	<0.001
Total cholesterol (mg/dl)	212 ± 38	217 ± 44	212 ± 37	0.170
LDL cholesterol (mg/dl)	131 (112–154)	134 (106–157)	131 (112–154)	0.833
Triglycerides (mg/dl)	103 (74–148)	132 (102–190)	100 (71–142)	<0.001
HDL cholesterol (mg/dl)	54 ± 12	50 ± 13	55 ± 12	<0.001
Low HDL-cholesterol (%)	21.4	35.2	19.4	<0.001
Lp(a) (mg/dl)	13.8 (5.8–32)	13.1 (4.8–29.3)	13.8 (6.0–33.1)	0.354
Lp(a) >46 mg/dl (%)	13.7	9.4	14.3	0.129
Fasting plasma glucose (mg/dl)	91 (82–101)	138 (116–177)	88 (82–97)	<0.001
HbA_1c_ (%)	5.4 (5.1–5.7)	6.8 (6.0–8.2)	5.3 (5.1–5.6)	<0.001
HOMA-IR	1.6 (1.0–2.7)	4.0 (2.7–6.6)	1.5 (1.0–2.3)	<0.001
Serum creatinine (mg/dl)	0.8 (0.7–0.9)	0.8 (0.7–1.0)	0.8 (0.7–0.9)	0.001

Results are expressed as mean ± SD or median (interquartile range) for variables with skewed distribution.

Subjects with Lp(a) >46 mg/dl were older than those with Lp(a) ≤46 mg/dl (50.0 ± 11.9 years vs. 47.7 ± 11.9 years, *p* = 0.034). The gender distribution, the percentage of postmenopausal women, and the percentage of women on hormone replacement therapy was not significantly different between groups with levels of Lp(a) ≤46 vs. >46 mg/dl.

**Figure 1:**
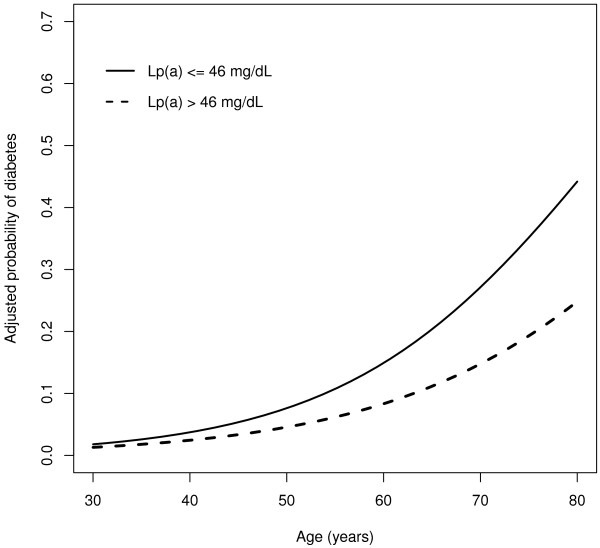
Adjusted probability of diabetes mellitus according to age and status of Lp(a) status.

As expected, most of the variables traditionally linked to diabetes were independently associated with the outcome in the multivariate logistic regression model: age (*p* < 0.001), MAP (*p* < 0.001), serum triglycerides (*p* = 0.001), and the interaction term between age and low HDL cholesterol (*p* = 0.007). In addition, there was a significant inverse association between diabetes and the interaction term age × Lp(a) >46 mg/dl (*p* = 0.048). As both HDL cholesterol and Lp(a) >46 mg/dl showed significant interactions with age, the association between age and diabetes was different in each of the four cohorts determined by the two factors (Table 2). Figure 1 shows the probability of diabetes according to age and Lp(a) level, adjusting for triglycerides, MAP, and HDL cholesterol.

**Table 2:** Results of multivariate logistic regression analysis

**Factor**	**Cohort**	**OR (95% CI)**
Age (per year)	Lp(a) ≤46 mg/dl/Normal HDL-C	1.075 (1.055 ; 1.095)
	Lp(a) >46 mg/dl/Normal HDL-C	1.062 (1.039 ; 1.085)
	Lp(a) ≤46 mg/dl/Low HDL-C	1.087 (1.066 ; 1.109)
	Lp(a) >46 mg/dl/Low HDL-C	1.074 (1.050 ; 1.098)
Mean arterial pressure	All subjects	1.060 (1.039 ; 1.081)
Log-Triglycerides	All subjects	2.317 (1.417 ; 3.787)

HDL-C: HDL cholesterol.

**Figure 2:**
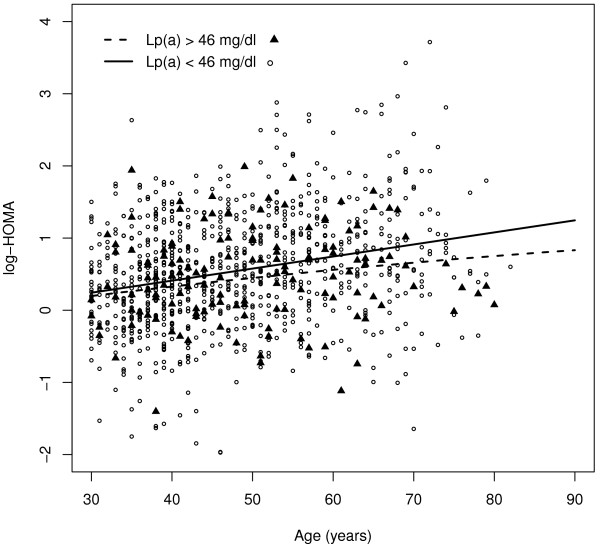
Parametric adjustment of HOMA-IR (log scale) for subjects with Lp(a) levels >46 mg/dl and subjects with Lp(a) levels ≤46 mg/dl.

Figure 2 shows the adjustment of log (HOMA-IR) according to our proposed parametric model for subjects with levels of Lp(a) >46 and for subjects with levels of Lp(a) ≤46 mg/dl. Predicted values of log (HOMA-IR) were higher in subjects with Lp(a) levels ≤46 mg/dl. The differences increased with the age, although the lower limit of the two-sided 95% confidence interval for the difference between predicted values of log (HOMA-IR) was greater than 0 across all ages.

## Discussion

The major finding in this population-based study was that after adjusting for established risk factors for diabetes (age, abdominal obesity, blood pressure, and serum levels of HDL cholesterol and triglycerides), the age-related increase in the probability of having diabetes was significantly lower in subjects with higher levels of Lp(a).

This was a cross-sectional study, so no causal associations can be inferred from the results. In theory, either diabetes by itself or any accompanying condition could contribute to increase Lp(a) levels over the lifespan of each individual. However, it is well established that Lp(a) concentrations are not significantly affected by environmental factors. Instead, most of the variance in plasma Lp(a) is determined genetically. Alternatively, because both Lp(a) and diabetes increase the risk of cardiovascular disease, mortality rates may be increased at earlier ages in subjects with both risk factors. This would result in a greater proportion of individuals with low levels of Lp(a) among older survivors with diabetes. However, this possibility is inconsistent with recent data showing that, in contrast to what is observed in the general population, plasma Lp(a) levels are not significantly associated with cardiovascular risk in patients with diabetes [18]. Therefore, a more plausible explanation for our findings is that the age-related rate of progression to diabetes is slower in subjects with high levels of Lp(a). This interpretation is in accordance with the results of the WHS, a large prospective study involving more than 26,000 healthy middle-aged US women, which found that the cumulative probability of incident type 2 diabetes was inversely correlated with basal Lp(a) levels [11]. However, in contrast with the WHS and the CCHS, our study found a threshold effect for Lp(a). Specifically, the age-related risk of diabetes was lower for subjects with Lp(a) levels above 46 mg/dl.

We also observed that values of HOMA-IR, a surrogate marker of insulin resistance were lower in subjects with Lp(a) levels >46 mg/dl, suggesting that extremely high levels of Lp(a) are associated with less resistance to insulin. Some previous population-based studies failed to demonstrate any relationship between insulin sensitivity and Lp(a) levels [19]. However, in their seminal investigations in the 1970s, Dahlën & Berg [20] found that, compared to controls with Lp(a) levels that were undetectable by agarose electrophoresis, older men with detectable Lp(a) levels showed higher levels of post-load blood glucose and lower levels of both fasting and post-load insulin. More recently, using the euglycemic clamp technique, Haffner et al [21] observed a positive correlation between insulin sensitivity and Lp(a) levels in normoglycemic men. In a large sample of subjects in the San Antonio Family Heart Study, the same authors demonstrated that Lp(a) levels are inversely correlated with fasting insulin levels and with insulin and glucose concentrations measured 2 hours after an OGTT [22]. Finally, several studies have shown a positive correlation between Lp(a) and HDL cholesterol and a negative correlation between Lp(a) and triglycerides [23], indicating that Lp(a) shows an inverse relationship with the atherogenic dyslipidemia characteristically associated with insulin resistance. There is no obvious explanation for this inverse correlation between Lp(a) and both diabetes and insulin resistance. Because Lp(a) levels are determined mainly by genetic mechanisms, one possibility is that genetic polymorphisms associated with increased levels of Lp(a) are in linkage disequilibrium with gene(s) that protect against insulin resistance.

## Conclusions

The present report suggests that the probability of having diabetes increases with age but that this increase is lower in subjects with serum levels of Lp(a) higher than 46 mg/dl. Further studies are needed to clarify the nature of the interaction between Lp(a) and the risk of diabetes.

## Abbreviations

CART, Classification and regression tree; CCHS, Copenhagen City Heart Study; HOMA-IR, Homeostasis Model Assessment for Insulin Resistance; Lp(a), Lipoprotein(a); MAP, Mean arterial pressure; OGTT, Oral glucose tolerance test; WHS, Women’s Health Study.

## Competing interests

The authors declare that they have no competing interests.

## Authors’ contributions

M.B. conceived the study and wrote the manuscript, P.S. performed the statistical analyses, N.P.-M. M.J.L.-M. and C.R.-P. researched the data and helped to draft the manuscript, and F.J.N. reviewed the manuscript and contributed to the discussion. All authors read and approved the final manuscript.
